# Accuracy of genomic selection and long‐term genetic gain for resistance to Verticillium wilt in strawberry

**DOI:** 10.1002/tpg2.20054

**Published:** 2020-09-24

**Authors:** Dominique D. A. Pincot, Michael A. Hardigan, Glenn S. Cole, Randi A. Famula, Peter M. Henry, Thomas R. Gordon, Steven J. Knapp

**Affiliations:** ^1^ Department of Plant Sciences University of California One Shields Avenue Davis CA 95616 USA; ^2^ United States Department of Agriculture 1636 E. Alisal Street Salinas CA 93905 USA; ^3^ Department of Plant Pathology University of California One Shields Avenue Davis CA 95616 USA

## Abstract

Verticillium wilt, a soil‐borne disease caused by the fungal pathogen *Verticillium dahliae*, threatens strawberry (*Fragaria* × *ananassa*) production worldwide. The development of resistant cultivars has been a persistent challenge, in part because the genetics of resistance is complex. The heritability of resistance and genetic gains in breeding for resistance to this pathogen have not been well documented. To elucidate the genetics, assess long‐term genetic gains, and estimate the accuracy of genomic selection for resistance to Verticillium wilt, we analyzed a genetically diverse population of elite and exotic germplasm accessions (*n* = 984), including 245 cultivars developed since 1854. We observed a full range of phenotypes, from highly susceptible to highly resistant: < 3% were classified as highly resistant, whereas > 50% were classified as moderately to highly susceptible. Broad‐sense heritability estimates ranged from 0.70–0.76, whereas narrow‐sense genomic heritability estimates ranged from 0.33–0.45. We found that genetic gains in breeding for resistance to Verticillium wilt have been negative over the last 165 years (mean resistance has decreased over time). We identified several highly resistant accessions that might harbor favorable alleles that are either rare or non‐existent in modern populations. We did not observe the segregation of large‐effect loci. The accuracy of genomic predictions ranged from 0.38–0.53 among years and whole‐genome regression methods. We show that genomic selection has promise for increasing genetic gains and accelerating the development of resistant cultivars in strawberry by shortening selection cycles and enabling selection in early developmental stages without phenotyping.

**Abbreviations**
BL: Bayesian Lasso
G‐BLUP: genomic best linear unbiased prediction
GEBV: genomic estimated breeding value
GWAS: genome wide association study
LMM: linear mixed model
LSM: least‐square mean
MAS: Marker‐assisted selection
MCCV: Monte Carlo cross‐validation
NPGS: National Plant Germplasm System
QTL: quantitative trait locus
RCB: randomized complete block
REML: restricted maximum likelihood
R‐gene: resistance gene
RKHS: Reproducing Kernel Hilbert Spaces
SNP: single nucleotide polymorphism
UCD: University of California Davis
VCG: vegetative compatibility group
WGR: whole‐genome regression.

## 1 INTRODUCTION

Verticillium wilt, a soil‐borne disease caused by the fungal pathogen *Verticillium dahliae* Klebahn, infects cultivated strawberry [*Fragaria* × *ananassa* (Weston) Duchesne ex Rozier] and numerous other economically important plants worldwide (Bhat & Subbarao, 1999; Fradin & Thomma, 2006; Pegg, 1984; Subbarao, Kabir, Martin, & Koike, 2007). This pathogen causes wilting and death in susceptible genotypes and was first positively identified on strawberry in the early 1900s (Thomas, 1932). Since the 1960s, soil fumigation with methyl bromide (MeBr) and other chemicals has been the principal method of managing the disease in conventional production systems, along with breeding for resistance (Njoroge, Kabir, Martin, Koike, & Subbarao, 2009; Strand, 2008; Wilhelm & Koch, 1956; Wilhelm & Paulus, 1980; Wilhelm, Storkan, & Wilhelm, 1974). Chemical fumigants suppress soil‐borne pathogen populations and reduce the incidence of soil‐borne diseases, thereby enabling highly predictable production without significant disease losses (Guthman, 2019; Holmes, Mansouripour, & Hewavitharana, 2020; Ristaino & Thomas, 1997; Subbarao et al., 2007; Wilhelm et al., 1974). The application of MeBr was banned by a global treaty and phased out between 2005 and 2016, prompting fundamental changes in management practices and the search for chemical and chemical‐free disease management alternatives (Holmes et al., 2020; Ristaino & Thomas, 1997). The uncertain future and efficacy of these alternatives and need for safe and effective solutions for organic production have reinforced the importance of breeding for resistance to *V. dahliae* and other soil‐borne pathogens. Our studies focused on assessing the progress in breeding for resistance to Verticillium wilt, the prevalence and strength of natural genetic resistance to Verticillium wilt in the octoploid gene pool, and the effectiveness of genome‐informed approaches for enhancing resistance to Verticillium wilt in cultivated strawberry.

*V. dahliae* causes disease in numerous economically important plants (Bolek et al., 2005; Bowen, Hough, & Varney, 1968; Fradin & Thomma, 2006; Hunter, Darling, Stevenson, & Cunningham, 1968; Pegg & Brady, 2002; Simko, Costanzo, Haynes, Christ, & Jones, 2004). Classic race‐specific resistance (*R*) genes have been discovered in several *V. dahliae* hosts, e.g., lettuce (*Lactuca sativa* L.; Hayes et al., 2011), tomato (*Solanum lycopersicum* L.; Fradin et al., 2009; Schaible, Cannon, & Waddoups, 1951), and others (Jansky, Rouse, & Kauth, 2004; Lynch, Kawchuk, Hachey, Bains, & Howard, 1997). The most well characterized of these confer innate immunity and recognize avirulence factors (effectors) in the pathogen (Dangl, Horvath, & Staskawicz, 2013; Hayes, Vallad, Qin, Grube, & Subbarao, 2007; Jones & Dangl, 2006; Pegg & Brady, 2002). The standard breeding practice in those species has been to deploy *R*‐genes through phenotypic or marker‐assisted selection (MAS), despite the inherent risks associated with uncertain durability of gene‐for‐gene resistance (Mundt, 2018; Poland & Rutkoski, 2016). The simple (gene‐for‐gene) solution to the problem of developing resistant cultivars has not been feasible in strawberry because *R*‐genes conferring resistance to Verticillium wilt have not been identified (Maas, Galletta, & Draper, 1989; Vining, Davis, Jamieson, & Mahoney, 2015). Large‐effect quantitative trait loci (QTL) have not been identified either (Antanaviciute et al., 2015; Cockerton et al., 2019). Consequently, phenotypic selection for quantitative resistance to this pathogen has previously been the only solution (Bringhurst, Hansche, & Voth, 1968; Darrow, 1966; Maas et al., 1989; Shaw, Gubler, Larson, & Hansen, 1996, 2010). With recent advances in the development of genomic resources and supporting information (Edger et al., 2019; Hardigan et al., 2020; Whitaker et al., 2020), strawberry is well positioned for the application of genome‐informed breeding approaches (Crossa et al., 2017; Poland & Rutkoski, 2016). Here, we explore the prospects for increasing genetic gains and accelerating the development of Verticillium wilt resistant cultivars through the application of genomic selection, under the assumption that resistance to this pathogen is genetically complex in cultivated strawberry (Antanaviciute et al., 2015; Cockerton et al., 2019; Shaw et al., 1996; Vining et al., 2015).

**Core Ideas**

- Genetic gains in breeding for resistance to Verticillium wilt have been negative over the last 165 years.
- Less than 3% of the germplasm accessions phenotyped were classified as highly resistant.
- The strongest sources of resistance were heirloom cultivars and ecotypes predicted to carry favorable alleles that are not found in modern cultivars.
- Genomic selection has significant potential to increase genetic gains for resistance to Verticillium wilt.

The heritability of resistance and genetic gains in breeding for resistance to Verticillium wilt have not been well documented in strawberry. From previous studies (reviewed by Vining et al., 2015), we hypothesized that the realized genetic gains in breeding for resistance to Verticillium wilt have been negligible (Antanaviciute et al., 2015; Cockerton et al., 2019; Maas et al., 1989; Shaw et al., 1996, 2010; Zurn et al., 2020). Several cultivars have been classified as resistant; however, none appear to be immune and few can be confidently described as highly resistant (Gordon & Subbarao, 2008; Maas et al., 1989; Shaw et al., 1996; Vining et al., 2015). The designation of resistance phenotypes has been particularly challenging because of inconsistencies across studies and the imprecision and ambiguity of qualitative phenotypic descriptors—no less than 11 qualitative phenotypic descriptors have been used to classify Verticillium wilt resistance phenotypes in strawberry (Maas et al., 1989; Vining et al., 2015). Because genetic variation for resistance to this pathogen is quantitative, resistance phenotypes can be more consistently and accurately described using ordinal or continuous phenotypic variables (Antanaviciute et al., 2015; Cockerton et al., 2019; Collado‐Romero, Mercado‐Blanco, Olivares‐García, & Jiménez‐Díaz, 2008, 2009; Gordon & Subbarao, 2008; Shaw et al., 1996).

To assess long‐term genetic gains in breeding for resistance to Verticillium wilt, we assembled and analyzed a genetically diverse population of elite and exotic germplasm accessions (*n* = 984). These included 876 *F*. × *ananassa* accessions developed in public breeding programs since 1854 and 99 phylogenetically and demographically diverse ecotypes of *F. chiloensis* (L.) Miller and *F. virginiana* Duchesne, the wild octoploid progenitors of *F*. × *ananassa* (Darrow, 1966). The *F*. × *ananassa* accessions included heirloom and modern cultivars previously identified as resistant or susceptible (Antanaviciute et al., 2015; Cockerton et al., 2019; Maas et al., 1989; Vining et al., 2015). These accessions were phenotyped for resistance to Verticillium wilt in field studies using plants artificially inoculated with a virulent race of *V. dahliae* (Vd9602) found in California (Gordon, Kirkpatrick, Hansen, & Shaw, 2006). To assemble a training population for a genomic selection study, we selected a diverse sample of 347 *F*. × *ananassa*, 20 *F. chiloensis*, and 21 *F. virginiana* accessions from the broader population of 984 accessions. Their selection was informed by previous studies of nucleotide diversity and population structure (Hardigan et al., 2018, 2020).

The studies described here were facilitated by the development of a chromosome‐scale octoploid reference genome and 50K single nucleotide polymorphism (SNP) genotyping array for *F*. × *ananassa* (Edger et al., 2019; Hardigan et al., 2020). The 50K SNP array was developed by targeting sub‐genome specific DNA sequences physically anchored to the octoploid reference genome (Hardigan et al., 2020). The training population (*n* = 388) was genotyped with the 50K SNP array, which yielded a dense, genome‐wide framework of sub‐genome specific DNA markers to support the genome‐wide association and genomic selection studies described in this paper (de los Campos, Sorensen, & Gianola, 2015; Evans et al., 2018; Goddard & Hayes, 2007; Hayes et al., 2009; Visscher, Hill, & Wray, 2008). Sub‐genome specific genotyping approaches are tractable and effective in strawberry because of the sheer abundance of DNA variants in elite and wild populations and exceptional frequency with which homeologous DNA sequences can be differentiated and aligned to the octoploid reference genome (Hardigan et al., 2020). Functionally diploid genotyping of DNA variants in allo‐octoploid (2n = 8x = 56) strawberry populations simplifies forward genetic studies and enables the application of straightforward diploid Mendelian and quantitative genetic approaches (Hardigan et al., 2020; Whitaker et al., 2020). Here we describe the accuracy of genomic selection for resistance to Verticillium wilt, estimates of long‐term genetic gains in breeding for resistance to Verticillium wilt, and sources of favorable alleles for resistance to Verticillium wilt that we predict are either rare or non‐existent in modern strawberry cultivars.

## 2 MATERIALS AND METHODS

### 2.1 Plant material and clonal propagation

The germplasm accessions (individuals) we studied have been maintained by asexual (clonal) propagation *ex situ* since their origin in public breeding programs or collection from wild habitats. Collectively, 984 accessions were screened for resistance to Verticillium wilt in our study (Supplemental Files S1; Supplemental Figure S1). The population included 65 *F*. × *ananassa* cultivars developed at UCD since 1935, 179 cultivars developed in other public breeding programs since 1854, 25 non‐UCD and 607 UCD *F*. × *ananassa* progeny developed from crosses between non‐inbred parents, 57 *F. chiloensis* ecotypes, and 42 *F. virginiana* ecotypes (Supplemental File S1; Supplemental Figure S1). The *F. chiloensis* and *F. virginiana* ecotypes were originally collected from habitats throughout their natural ranges in North and South America (Staudt, 1988, 1999). We obtained source plants (clones) of these accessions from the UCD Strawberry Germplasm Collection or the United States Department of Agriculture Agricultural Research Service National Plant Germplasm System (USDA‐ARS NPGS) National Clonal Germplasm Repository, Corvallis, OR (https://www.ars-grin.gov/). The geographic origin, year of origin (birth year), country of origin, geographic coordinates, and taxonomic classifications, in addition to UCD identification (UCD ID) numbers for UCD accessions (*n* = 673) or plant introduction (PI) numbers for USDA accessions (*n* = 311), are documented in Supplemental File S1. Clonally propagated plants (daughter plants) of each accession were produced in high‐elevation (1,294 m) nurseries in Dorris, CA from mother plants produced in low‐elevation (41 m) nurseries in Winters, CA. The propagation of plant material was identical in both years (2016 and 2017): mother plants were harvested from the low‐elevation nursery in January, stored at −3.5 °C, and planted in the high‐elevation nursery in April. Clones were harvested in October and stored at 3.5 °C for two to three weeks before pathogen inoculation and planting.

### 2.2 Pathogen source material and inoculum preparation

Vd9602, the virulent *V. dahliae* isolate used in our study, was originally isolated from strawberry plants growing in a commercial nursery in Macdoel, California (Gordon et al., 2006). This isolate was genotyped for a species‐specific locus with sufficient resolution to distinguish most vegetative compatibility groups (VCG) characterized for *V. dahliae* (Collado‐Romero et al., 2008). DNA was first extracted with the Prepman Ultra Sample Preparation Reagent (ThermoFisher Scientific, LLC) as per manufacturer's instructions. PCR reaction mixtures contained 20 ng gDNA, 1x OneTaq master mix with standard buffer (New England Biolabs, Inc.), and 400 nm each of primers DB19 (5′‐CGG TGA CAT AAT ACT GAG AG‐3′) and DB22 (5′‐GAC GAT GCG GAT TGA ACG AA‐3′) (Collado‐Romero et al., 2008). Thermocycler parameters were: 94 °C for 5 min for an initial denaturation followed by 35 cycles of 1 min at 94 °C, 1 min at 55 °C, 1 min at 72 °C, and a final extension of 6 min at 72 °C (Collado‐Romero et al., 2008). PCR products were visualized on a 1.5% agarose gel in 1X TAE buffer with GelRed (Biotium, LLC) and purified with AMPure XP beads (Beckman Coulter Life Sciences, Inc.) as per manufacturer's instructions. Sanger sequencing of PCR products was conducted by Eton Biosciences, Inc. to 4x depth. Reads were assembled in Geneious Prime (version 2019.0.4) and compared with type sequences from isolates V640I (VCG 4A), V559I (VCGX), V803I (VCG 4B), V357I (VCG 2B), 131‐M (VCG 4A), and 70‐21 (GenBank accessions: DQ266244.1‐DQ266249.1) (Collado‐Romero et al., 2008). Vd9602 shares a species‐specific haplotype with VCG4B isolates—the latter fall within *V. dahliae* subclade II‐1 (Fan et al., 2018; Milgroom, del Mar Jimenez‐Gasco, Garcia, Drott, & Jimenez‐Diaz, 2014). *V. dahliae* subclades II‐1 and II‐2 contain virulent isolates. Clear pathogen genotype by host interactions have not been observed from isolates in this clade (Cockerton et al., 2019; Fan et al., 2018; Zeise & von Tiedemann, 2002). VCG4B isolates are frequently recovered from diseased strawberry plants (Bhat & Subbarao, 1999; Zeise & von Tiedemann, 2002), especially in coastal California (Atallah, Hayes, & Subbarao, 2011). The species‐specific sequence for the Vd9602 isolate was deposited in GenBank (MN813047).

To produce spores for artificially inoculating strawberry plants, the pathogen was grown on potato dextrose agar under continuous fluorescent lighting at room temperature, similar to the protocol described by Shaw et al. (1996). Spores were freed from the surface by adding sterile, deionized water to the growth plates and scraping the medium with a sterile glass slide. The resulting suspension was filtered through two layers of sterilized cheesecloth to remove hyphae. Spore densities were estimated using a haemocytometer and diluted with sterile, deionized water to a final density of 1 × 10^6^ spores ml^−1^. Spore suspensions were prepared one day prior to planting and stored at 4 °C overnight. The suspension was shaken to re‐suspend spores before inoculating bare‐root plants.

### 2.3 Field experiments

Accessions were grown and phenotyped for resistance to Verticillium wilt in field experiments at the UCD Plant Pathology Research Farm in Davis, CA, where the soil type is a Yolo loam (https://websoilsurvey.sc.egov.usda.gov/). Strawberries had not been previously grown on the field sites selected for our studies. Site preparation, inoculation of plants with the pathogen, planting, fertilization, irrigation, and other management practices were identical in both years. Fields were pre‐plant flat‐fumigated with a chloropicrin‐based fumigant (Pic‐Clor 60, Cardinal Professional Products, Woodland, CA; 500 lb ac^−1^) and sealed with a totally impermeable film tarp for one‐week post‐fumigation. The field sites were subsequently prepared for planting by creating 15.25 cm high raised beds with 76.2 cm of spacing between beds center‐to‐center. Subsequent to installing drip irrigation, beds were covered with black plastic mulch. Sub‐surface drip irrigation was applied as needed to maintain adequate soil moisture throughout the growing season. Fertilization was done via injection through the drip system with approximately 198 kg ha^−1^ of nitrogen applied over the growing season.

The roots of each clone (bare‐root plant) were submerged in the *V. dahliae* Vd9602 inoculum suspension (1 × 10^6^ spores ml^−1^) for 7–8 minutes before transplanting into a single row in the middle of planting beds. The spacing was 30.5 cm between plants within rows. The planting dates were October 31, 2016 and October 23, 2017. We arranged accessions in square lattice experiment designs with four single‐plant replications per accession (Hinkelmann & Kempthorne, 2005). There were four complete blocks (replications) in both years with incomplete blocks arranged in randomized complete blocks (RCBs). We used a 31 × 31 square lattice with 31 accessions/incomplete block × 31 incomplete blocks (961 accessions) in 2016–2017 and a 22 × 22 square lattice with 22 accessions/incomplete block × 22 incomplete blocks (484 accessions) in 2017–2018. The R package *agricolae* (de Mendiburu, 2017) was used to assign accessions to incomplete blocks and randomize accessions within incomplete blocks. The accessions in the genomic selection training population (*n* = 388) were phenotyped in both years.

### 2.4 Disease resistance phenotyping

Each plant was phenotyped for resistance to Verticillium wilt by visually scoring the severity of symptoms on a 1 to 9 scale, where 1 = symptomless and 9 = complete dieback. Symptom severity was estimated using a combination of stunting, wilting, chlorosis, and dieback, as described by Gordon et al. (2006). Phenotyping was initiated as soon as symptoms appeared and continued on a bi‐weekly basis from May 30 to July 26 in 2017 and May 7 to June 29 in 2018. This corresponded to 30 to 38 weeks post‐inoculation in 2017 and 28 to 36 weeks post‐inoculation in 2018. We used the progression of disease symptoms to determine when to terminate phenotyping. Symptoms had fully progressed and were most extreme among resistant and susceptible checks in the final time point in each year (38 weeks in 2017 and 36 weeks in 2018). Statistical analyses were applied to phenotypic data collected at these final timepoints.

### 2.5 SNP marker genotyping

DNA was isolated from newly emerged leaves harvested from field grown plants. Leaf samples were collected into 1.1 ml tubes or coin envelopes and freeze‐dried in a Benchtop Pro (VirTis SP Scientific, Stone Bridge, NY). We placed 0.2 g of dried leaf tissue/sample into wells of 2.0 ml 96‐well deep‐well plates. These tissue samples were ground using stainless steel beads in a Mini 1600 (SPEX Sample Prep, Metuchen, NJ). Genomic DNA (gDNA) was extracted from powdered leaf samples using the E‐Z 96 Plant DNA Kit (Omega Bio‐Tek, Norcross, GA, USA) according to the manufacturer's instructions. To enhance the quality and yield of the DNA and reduce polysaccharide carry‐through, the protocol was modified by adding Proteinase K to the lysis buffer to a final concentration of 0.2 mg ml^−1^ and extending lysis incubation for 45 min at 65 °C. Once the lysate separated from the cellular debris, RNA was removed by adding RNase A and incubating the mixture at room temperature for 5 min. before a final spin down. To ensure high DNA yields, the sample was incubated at 65 °C for 5 min following the addition of elution buffer. DNA quantification was performed using Quantiflor dye (Promega, Madison, WI) on a Synergy HTX (Biotek, Winooski, VT).

Training population accessions were genotyped with the Affymetrix 50K SNP Axiom Array (Hardigan et al., 2020) on a GeneTitan HT Microarray System at Affymetrix (Santa Clara, CA) using gDNA samples that passed quality and quantity control standards. SNP genotypes were automatically called with the Affymetrix Axiom Analysis Suite software (v1.1.1.66, Affymetrix, Santa Clara, CA) followed by manual curation. All samples had a call‐rate greater than 90% (< 10% missing data) and were retained for further analysis. The Affymetrix Axiom Analysis Suite and custom R scripts were used to identify and select SNP markers with well separated codominant genotypic clusters (SNP markers with non‐overlapping signals for each of the three genotypes). The filtering and selection process yielded 35,144 high‐quality codominant SNP markers for subsequent statistical analyses.

### 2.6 Statistical analyses

We used the R package *lme4* for linear mixed model (LMM) analyses (Bates, Machler, Bolker, & Walker, 2015). The data were analyzed using LMMs for square lattice and randomized complete blocks (RCB) experiment designs. We did not observe an increase in efficiency for lattice relative to RCB experiment designs in either year; hence, the data were analyzed using LMMs for RCB experiment designs (incomplete blocks were ignored) with random year, accession, accession × year, and residual effects (Hinkelmann & Kempthorne, 2005). Variance components for these effects were estimated using restricted maximum likelihood (REML). Heritability on a clone‐mean basis (*H* ^2^ = V_G_/V_P_) was estimated from REML estimates of the accession (V_G_), accession × year (V_G×Y_), and residual (V_E_) variance components, where V_P_ = V_G_ + V_G×Y_/*y* + V_E_/*ry, y* is the number of years, and *r* is the harmonic mean of the number of replications per accession: *r* was less than four because of random missing data, e.g., plants that did not survive planting or establishment. The harmonic means were *r* = 3.67 in 2017, 3.77 in 2018, and 7.65 between years. The least‐square means (LSMs) for accessions were estimated using the R package *emmeans* (Lenth, 2016, 2018) and used as dependent variables (phenotypic observations) for the genome‐wide association study (GWAS) and genomic prediction study. The phenotypic correlation between 2017 and 2018 LSMs for accessions was estimated using the R function *cor.test* (R Core Team, 2018).

### 2.7 Genome‐wide association study

The additive genetic relationship matrix (G) was estimated for training population accessions from SNP marker genotypes using the *A.mat()* function in the R package *rrBLUP* (Endelman, 2011). SNP marker genotypes were recoded 1, 0, and −1 to estimate G (VanRaden, 2008). The genetic structure of the training population was investigated using hierarchical clustering and principal components analysis of the G matrix as described by Crossa et al. (2014). To correct for population structure and genetic relatedness in GWAS analyses, a Q + K linear mixed model was used where Q is the population stratification structure matrix and K is the kinship matrix (Kang et al., 2008; Yu et al., 2006). We substituted G for K. The first three principal components from eigenvalue decomposition of the K matrix were incorporated into the Q+K model. GWAS statistics were estimated within and between years from the LSMs for accessions using the *GWAS()* function of *rrBLUP* (Endelman, 2011). Genomic inflation factors (Freedman et al., 2004) after correcting for population structure were 1.03 for 2017, 1.00 for 2018, and 1.02 between years. We used permutation tests to calculate significance thresholds for GWAS hypothesis testing (Gao, 2011). The permutation significance thresholds were 3.3 × 10^−6^ (−log_10_ *p*‐value = 5.48) for 2017, 9.5 × 10^−6^ (−log_10_ *p*‐value = 5.02) for 2018, and 4.3 × 10^−6^ (−log_10_ *p*‐value = 5.37) for 2017 and 2018 combined.

### 2.8 Genomic selection and genomic heritability estimation

Genomic‐estimated breeding values (GEBVs) were estimated using genomic best linear unbiased prediction (G‐BLUP; Endelman, 2011), the Bayesian Lasso (BL; Park & Casella, 2008), and Reproducing Kernel Hilbert Spaces Regression (RKHS; Gianola & van Kaam, 2008). The input for these analyses were LSMs for accessions within and between years. Separate analyses were performed within and between years using the complete training population (347 *F*. × *ananassa*, 20 *F. chiloensis*, and 21 *F. virginiana* accessions) and *F*. × *ananassa* accessions only (*n* = 347).

GEBVs were estimated using three whole‐genome regression methods by assuming that the dependent variable distributions were normal (continuous) or categorical (ordinal) (Pérez & de los Campos, 2014). G‐BLUP analyses for the continuous model were performed using the *kin.blup()* function in the R package *rrBLUP* with the G matrix as input (Endelman, 2011; Endelman & Jannink, 2012). The G‐BLUP analysis for the ordinal response variable and BL and RKHS analyses for the continuous and ordinal response variables were performed using the R package *BGLR* with default parameter settings (Pérez & de los Campos, 2014). The kernel for RKHS was determined using the multi‐kernel averaging method (de los Campos, Gianola, Rosa, Weigel, & Crossa, 2010). For analyses where disease resistance phenotypes were treated as continuous response variables, the ‘response_type = continuous’ command in *BGLR* was used along with default settings for residual weights. For analyses with disease resistance phenotypes were treated as categorical (ordinal) response variables, the ‘response_type = ordinal’ command in *BGLR* was used. G‐BLUP, BL, and RKHS analyses for the ordinal model were performed using the probit link function implemented in *BGLR* (Pérez & de los Campos, 2014). Collectively, 54 analyses were performed for the continuous and ordinal response variables (108 analyses in total) and yielded GEBV estimates for calculating genomic prediction accuracies. We performed all possible combinations of analyses within and between years: data from 2017 were used to predict 2017, 2018, and between years, data from 2018 were used to predict 2017, 2018, and between years, and data between years were used to predict 2017, 2018, and between years.

To estimate the accuracy of genomic predictions, we used Monte Carlo cross‐validation (MCCV) with *k* = 10,000 replications generated by randomly splitting accessions into training (80%) and validation (20%) subsets (Burman, 1989; Molinaro, Simon, & Pfeiffer, 2005; Poland & Rutkoski, 2016). The accuracy of genomic selection (*r_GS_*) was estimated as the mean of correlations between observed phenotypes (*ȳ*) and GEBVs among the 10,000 replications, where the *ȳ* are LSMs for accessions in the training population. We estimated the accuracy of a genomic selection relative to phenotypic selection using *r_E_* = *r_GS_*/*h*, where *h* is the square root of narrow‐sense genomic heritability (*h* ^2^) (Crossa et al., 2014). Genomic selection accuracies were estimated for *F. × ananassa* accessions only (*n* = 347) and for the entire training population (*n* = 388). Cross‐validations were performed within and between years in all combinations, as outlined above. Narrow‐sense genomic heritability was estimated from the mean of 10,000 MCCV‐generated REML estimates of the variance components from G‐BLUP (Endelman, 2011; Mathew, Léon, & Sillanpää, 2018).

## 3 RESULTS AND DISCUSSION

### 3.1 Heritability of resistance to Verticillium wilt

**FIGURE 1:**
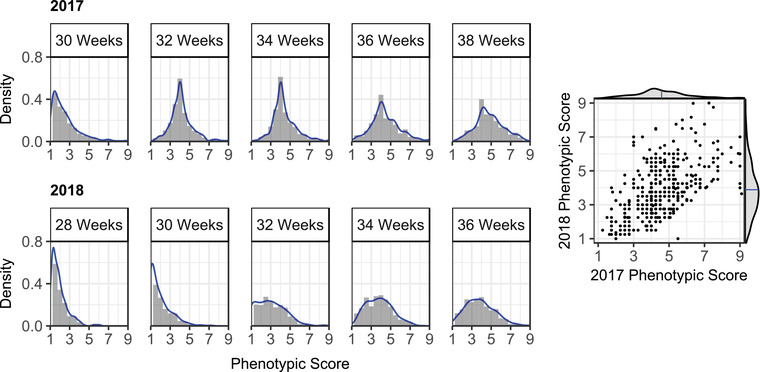
Verticillium wilt resistance distributions for strawberry populations phenotyped in 2017 and 2018 field experiments in Davis, California using a 1 to 9 visual rating scale, where 1 = symptomless and 9 = complete die‐back. The severity of symptoms (stunting, wilting, chlorosis, and dieback) increased as disease severity scores increased. Histograms are shown for least square means (LSMs) estimated from four clonal replications/accession over multiple timepoints: 893 accessions were phenotyped in 2017 (upper panel) and 479 accessions were phenotyped in 2018 (lower panel). LSMs are shown in the scatterplot (far right panel) for training population accessions (*n* = 388) phenotyped at the final timepoints in 2017 and 2018

We observed significant genotypic and phenotypic variability for resistance to Verticillium wilt among accessions in the complete study population (*n* = 984) and genomic selection training population (*n* = 388)—accessions in the latter were phenotyped in both years, whereas the other 596 accessions were phenotyped in one year only (Figure 1; Supplemental File S1). The severity of symptoms increased over the eight‐week phenotyping period and plateaued at the final timepoints in both years. The least square means (LSMs) for accessions were approximately normally distributed at the final timepoints in each year, with accessions spanning the entire range from highly resistant (score = 1) to highly susceptible (score = 9). Symptoms were more severe in 2017 than 2018, which resulted in greater phenotypic separation among accessions in 2017 than 2018 and an increase in the number of accessions in the resistant range (≤3) in 2018 (Figure 1). The mean disease score for the training population was significantly lower in 2018 (3.7) than 2017 (4.2) (*p* ≤ .001). The genotype‐by‐year interaction was significant (*p* ≤ .001); however, disease scores were positively correlated between years (*r* = 0.54; *p* ≤ .001; Figure 1). The broad‐sense heritability estimates were 0.71 in 2017 (*n* = 893) and 0.70 in 2018 (*n* = 479). When estimated from training population accessions only (*n* = 388), the broad‐sense heritability estimates were 0.73 in 2017, 0.71 in 2018, and 0.76 between years, whereas the narrow‐sense genomic heritability estimates were 0.33 in 2017, 0.42 in 2018, and 0.45 between years.

### 3.2 Genetic gain for resistance to Verticillium wilt

**FIGURE 2:**
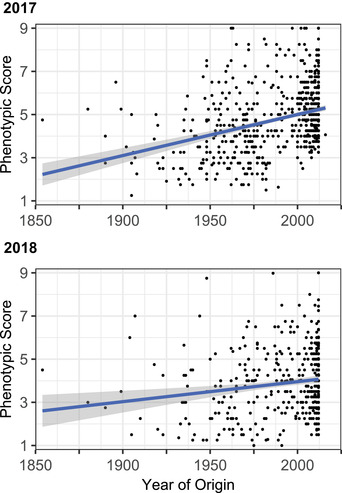
Verticillium wilt resistance phenotypes (least square means) for 856 strawberry accessions originating between 1854 and 2016. The linear regressions of phenotypic score on year of origin are shown for 768 accessions phenotyped in 2017 (upper panel; adjusted *R* ^2^ = 0.12; *p* ≤ .001) and 431 accessions phenotyped in 2018 (lower panel; adjusted *R* ^2^ = 0.03; *p* ≤ .001). The blue lines are slopes for linear regressions of phenotypic score on year of origin with 95% confidence intervals shown as gray bands

Our phenotypic observations suggest that resistance to Verticillium wilt has decreased over the last 165 years of strawberry breeding. When the resistance phenotypes of *F. × ananassa* accessions developed since 1854 were compared, we found that susceptibility to Verticillium wilt had increased over time—the genetic gain was negative (Figure 2; Supplemental File S1). Correlations between resistance phenotype and year of origin were 0.35 in 2017 (*p* ≤ .001), 0.18 in 2018 (*p* ≤ .001), and 0.31 between years (*p* ≤ .001). The linear regression of resistance score on year of origin, while statistically significant (*p* ≤ 0.001) and positive, was nevertheless only weakly predictive because of the wide dispersion of phenotypic observations over different years of origin (adjusted *R* ^2^ = 0.03–0.12; Figure 2)—a positive slope equates to a negative genetic gain because resistance to Verticillium wilt decreases as disease severity scores increase on the ordinal scale applied in our study. From approximately 1950 onward, the frequency of susceptible accessions increased. Moreover, the frequency of accessions with disease scores in the resistant range (<3) decreased from 1987 onward—that trend was more pronounced in 2017 than 2018 (Figure 2).

To explore these trends in greater depth, we estimated genetic gains for 178 non‐UCD cultivars developed since 1854 (Supplemental File S1). This sample included every publicly available non‐UCD cultivar that we could acquire. The resistance scores for those cultivars ranged from 1.5–9.0 in 2017 and 1.0–8.8 in 2018 (Supplemental File S1). The correlations between mean disease score and year of origin for those cultivars were not significantly different from zero (no genetic gain) in 2017 (*r* = −0.02; *p* = .84) or 2018 (*r* = −0.16; *p* = .17).

**FIGURE 3:**
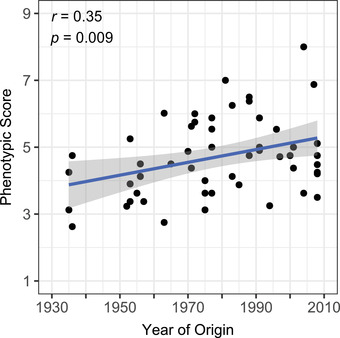
Verticillium wilt resistance phenotypes for strawberry cultivars developed at the University of California, Davis from 1935 to 2011. The linear regression of phenotypic score on year of origin is shown for 57 cultivars. The phenotypic scores are least square means for disease severity scores estimated from four clonal replicates/year in 2017 and 2018 field studies (adjusted‐*R* ^2^ = 0.10; *p* = 0.009). The blue line is the slope for the linear regression of phenotypic score on year of origin with 95% confidence intervals shown as gray bands

Finally, we estimated genetic gains for 65 of 69 UCD cultivars developed since 1935 (Figure 3; Supplemental File S1). The resistance scores for those cultivars ranged from 2.3–9.0 in 2017 and 1.8–8.0 in 2018. Consistent with the trends observed for non‐UCD cultivars and the broader population (Figure 2), the genetic gain among UCD cultivars was negative (Figure 3). When resistance score was regressed on year of origin, we found that the susceptibility of UCD cultivars had increased over time (*b* = 0.019 ± 0.007; *p* = .009; *r* = 0.35; Figure 3). The phenotypic increase (decrease in resistance) over the 76‐year period was estimated to be 1.4 on the 1 to 9 ordinal scale. We partly attributed the increase in susceptibility to an increase in the number of susceptible cultivars that were released subsequent to the advent of soil fumigation in the late 1950s (Wilhelm & Koch, 1956; Wilhelm & Paulus, 1980; Wilhelm et al., 1974). UCD cultivars developed before the introduction of soil fumigation (1935–1957) had resistance scores in the more resistant range (2.6–5.3), whereas UCD cultivars developed between 1960 and 2011 had resistance scores across the phenotypic range (2.7–8.0), with approximately half of the cultivars falling in the upper (more susceptible) half of the phenotypic distribution (Figure 3). Not only was disease pressure lessened by the application of soil fumigants starting in the late 1950s, but the effectiveness of soil fumigation meant that even highly susceptible cultivars could be grown with predictable results (Wilhelm & Koch, 1956; Wilhelm & Paulus, 1980; Wilhelm et al., 1974).

The results of our study contradict a report by Shaw et al. (2010) that genetic gains in breeding for resistance to Verticillium wilt had been significant in the UCD population between 1987 and 2005. We could not duplicate their study because the accessions they tested were not identified; however, we tested 160 UCD accessions developed between 1987 and 2005—this sample included every accession from that period preserved in the UCD Strawberry Germplasm Collection (Supplemental File S1). The resistance phenotypes of those accessions ranged from 2.5 to 9.0 in 2017 and 1.0 to 6.8 in 2018. The correlation between resistance score and year of origin was not significantly different from zero in either year of our study—*r* = −0.09 (*p* = .35) in 2017 and *r* = −0.17 (*p* = .19) in 2018 (Supplemental File S1). Hence, we did not observe a statistically significant genetic change in the UCD population between 1987 and 2005. The negative genetic gains from 1927 to present in the UCD population highlight the persistence of genetic variation (favorable and unfavorable alleles) for resistance to Verticillium wilt and an increase in the frequency of susceptible individuals and unfavorable alleles in the reference population over time, as opposed to a lack of progress in breeding for resistance (Supplemental File S1; Figure 1, 2, 3).

### 3.3 Verticillium wilt resistance among heirloom and modern cultivars

Although genetic gains have been negative, several moderately to highly resistant cultivars (with disease scores in the 1 ≤ *ȳ* ≤ 3 range) have been developed over the last 165 years of breeding (Figure 2; Supplemental File S1). Fewer resistant cultivars have been developed since the 1960s when soil fumigants were introduced (Wilhelm & Paulus, 1980; Figure 2; Supplemental File S1). Cultivars developed between 1854 and 1930, a period that pre‐dated the discovery of Verticillium wilt in strawberry (Thomas, 1932), were weakly to strongly resistant—approximately 50% had disease scores in the resistant (1 to 3) range and 60% had disease scores below the population mean (were in the lower half of the phenotypic distribution) in both years of the study (Supplemental File S1). This suggests that these heirloom cultivars (the oldest preserved in public germplasm collections) emerged from breeding programs where natural Verticillium wilt pressure was present (Maas et al., 1989; Wilhelm & Paulus, 1980).

Nine of the 10 most resistant accessions (the upper 1%) in our study were *F. × ananassa* cultivars or other accessions that originated between 1905 and 1989 in high latitude breeding programs, from Geneva, New York (42.9° N lat) to Sitka, Alaska (57.1° N lat) in North America and Acona, Italy (43.6° N lat) to Wageningen, the Netherlands (51.6° N lat) in Europe (Supplemental File S1; Supplemental Figure S1). The three most resistant cultivars were Empire (*ȳ* = 1.5), Beaver Belle (*ȳ* = 1.5), and Jewel (*ȳ* = 1.6). The two most resistant accessions were ORUS 4816 and Sitka, both of which originated along the coast of northwestern North America. The parents of ORUS 4816 (Totem × Benton), which was the single‐most resistant accession (*ȳ*_17 _= 1.0, where *ȳ*_17_ is the LSM for 2017), were moderately resistant (Totem; *ȳ*_17_ = 2.8) or weakly susceptible (Benton; *ȳ*_17_ = 4.5) (Supplemental File S1). Sitka, the second‐most resistant accession in our study (*ȳ* = 1.4), appears to be a hybrid between an unknown Alaskan *F. chiloensis* subsp. *pacifica* Staudt ecotype and an unknown *F. × ananassa* accession. CA 1499—an Alaskan *F. chiloensis* subsp. *pacifica* ecotype—was the single‐most resistant wild ecotype in our study (*ȳ* = 1.5). Hence, coastal Alaskan *F. chiloensis* subsp. *pacifica* ecotypes and their hybrid offspring warrant further study and might harbor favorable alleles that are either absent or rare in modern cultivars (Supplemental File S1).

The most resistant UCD cultivars identified in our study were developed between 1935 and 1975: Tahoe (*ȳ* = 2.6), Tufts (*ȳ* = 2.8), Shasta (*ȳ* = 3.1), Selva (*ȳ* = 3.1), and Wiltguard (*ȳ* = 3.2) (Supplemental File S1). The most resistant UCD cultivars developed since 1994 were UCD Moxie (*ȳ* = 3.2) and Camino Real (*ȳ* = 3.3). None of these cultivars were significantly more resistant than ORUS 4816, Sitka, Empire, or Beaver Belle (*p* ≤ 0.001). Hence, breeding over the last 30 years has not increased Verticillium resistance beyond that of early UCD cultivars (1927‐1975) or the most resistant non‐UCD cultivars. We could not acquire plants of patent‐protected private and public sector cultivars for inclusion in our studies; however, Ivors et al. (2018) and Ivors, Fernandez, Brantley, and Holmes (2017) screened approximately 90 cultivars from UCD, the University of Florida, and four private sector breeding programs for resistance to Verticillium wilt in common garden experiments in San Luis Obispo, CA, in 2017 and 2018. Similar to the trends observed in our study, disease symptoms were more severe in 2017 than 2018. Collectively, public sector cultivars were more resistant than private sector cultivars in both years (Ivors et al., 2017, Ivors et al., 2018). The disease mortality percentage ranges were 0.0–38.6% for public sector cultivars and 3.3–78.2% for private sector cultivars in the 2017 study and 0.0–20.1% for public sector cultivars and 0.0–33.1% for private sector cultivars in the 2018 study. The Verticillium wilt resistance rankings for the UCD cultivars we included in the Ivors et al. (2017) and Ivors et al. (2018) studies were virtually identical to those observed in our studies. This suggests that the phenotypic ranges, genetic gains, and other trends observed in our studies accurately reflect the global picture in strawberry.

Cultivars developed at lower latitudes (< 30° N) were significantly less resistant (*p* ≤ .001) than cultivars developed at higher latitudes (> 47° N) (Supplemental Figure S2). The phenotypic ranges in the 2017 study were 4.0–7.8 for eight cultivars developed at lower latitudes (< 30° N) and 1.6–5.3 for 49 cultivars developed at higher latitudes (> 47° N). Collectively, cultivars developed in Europe (5° E to 19° W longitudes) were more resistant than cultivars developed in other parts of the world—the phenotypic range for European cultivars was 1.5 to 5.3 (Supplemental File S1; Supplemental Figure S2). Conversely, cultivars developed in Eastern Asia (121 to 141° W longitudes) were moderately to highly susceptible with resistance phenotypes in the 3.3–7.8 range, excluding the cultivar Morioka 17 (*ȳ*_17_ = 2.8). The prevalence of resistant accessions from high latitude habitats and breeding programs might stem from greater natural disease pressure in perennial production systems and environments optimum for pathogen survival and disease development, e.g., soil temperatures in the 21–27 °C range and cool overcast days interspersed with warm clear days (Pegg & Brady, 2002; Schnathorst, 1981).

### 3.4 Verticillium wilt resistance among wild octoploid ecotypes

To assess the frequency and strength of resistance among the wild octoploid progenitors of *F. × ananassa*, we screened 33 South American *F. chiloensis*, 25 North American *F. chiloensis*, and 42 *F. virginiana* ecotypes native to habitats across the natural geographic ranges of these species: 27.0° to 64.9° N in the northern hemisphere and −1.3° to −47.1° S in the southern hemisphere (Staudt, 1988, 1999; Supplemental Figure S2; Supplemental File S1). Strong resistance to Verticillium wilt (*ȳ* ≤ 2) was less common among the wild octoploid ecotypes (1/41 = 0.024) than among heirloom and modern cultivars (15/129 = 0.116) (Supplemental File S1). Amongst the 30 most highly resistant accessions identified in the 2017 study (upper 3.4% of the population), six were wild octoploid ecotypes. The only wild ecotype classified as highly resistant in the 2018 study was CA‐1499, which was also highly resistant in the 2017 study. Of these six wild ecotypes, five were native to northern latitudes in North America, from Clay Butte, WY (45.0° N latitude) to Auke Lake, AK (58.4° N latitude), consistent with the trend observed among the *F. × ananassa* accessions we studied (Supplemental File S1; Supplemental Figure S2). One of the six was 2‐CUC‐1A (PI 616529), a wild *F. chiloensis* ecotype from South America. None of the highly resistant wild ecotypes identified in our study were found in *F*. × *ananassa* pedigree records (unpublished data); hence, they might carry favorable alleles that are either rare or non‐existent in historically and commercially important germplasm.

Of the 33 *F. chiloensis* ecotypes from South America, 94% were susceptible to Verticillium wilt with disease severity scores in the 4.0–9.0 range in 2017 and 3.3–6.7 range in 2018 (Supplemental File S1). Of the six South American *F. chiloensis* ecotypes phenotyped in both years, none were highly resistant (3.6 ≤ *ȳ* ≤ 7.4). PI 616529 was the most resistant South American *F. chiloensis* ecotype in both years (*ȳ* = 3.6). Collectively, South American *F. chiloensis* ecotypes were significantly more susceptible than northern hemisphere *F. chiloensis* and *F. virginiana* ecotypes (*p* ≤ 0.001).

### 3.5 Genetic structure of the training population

**FIGURE 4:**
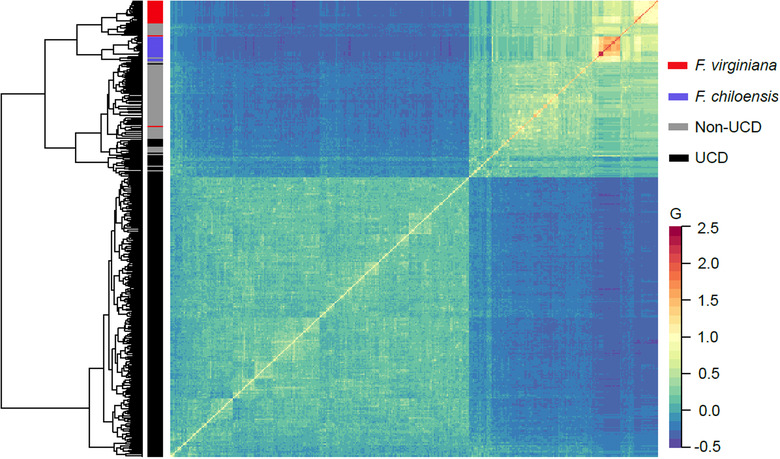
Additive genetic relationship matrix for 388 strawberry accessions estimated from the genotypes of 35,144 SNP markers assayed with a 50K array. Hierarchical clustering was used to order rows of the matrix and construct the dendrogram. *F. × ananassa* accessions are indicated by their origin (UCD or non‐UCD)

The additive genetic relationship matrix among training population accessions (*n* = 388) was estimated from the genotypes of 35,144 SNP markers distributed throughout the octoploid strawberry genome (Hardigan et al., 2020; Figure 4; Supplemental Figure S3). We observed a population structure consistent with earlier studies, with accessions falling into one of four groups: UCD *F. × ananassa*, non‐UCD *F. × ananassa*, *F. chiloensis*, and *F. virginiana* (Hardigan et al., 2018, 2020; Figure 4). The non‐UCD group was comprised of cultivars sampled from geographically diverse public breeding programs worldwide (Supplemental File S1). The earliest non‐UCD cultivars (1854–1930) clustered more closely with *F. virginiana* than more contemporary non‐UCD cultivars (1930–present). We observed a similar pattern between early (1930–1970) and modern (1970–present) UCD cultivars. This population structure was known *a priori* (Hardigan et al., 2018, 2020) and guided the selection of accessions for inclusion in the training population.

### 3.6 Genome‐wide association study

**FIGURE 5:**
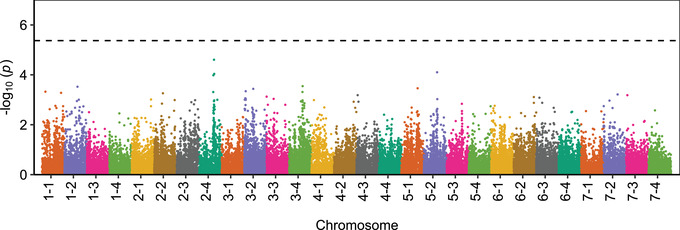
Manhattan plot displaying GWAS‐estimated −log_10_(*p*‐values) for Verticillium wilt resistance phenotypes in a training population comprised of 347 *F. × ananassa*, 20 *F. chiloensis*, and 21 *F. virginiana* accessions. GWAS statistics were estimated for resistance phenotypes between years using physical positions in an octoploid reference genome and 35,144 array‐genotyped SNPs. The significance threshold (dashed horizontal line) was calculated by permutation (−log_10_ *p*‐value = 5.37)

To lay the foundation for the genomic selection study and search the genome for significant marker‐trait associations, we applied GWAS to Verticillium wilt phenotypes in the training population. Statistically significant marker‐trait associations were not identified anywhere in the genome within or between years (Figure 5; Supplemental Figure S4). We concluded that genetic variation for resistance to Verticillium wilt was quantitative and not affected by any large‐effect loci in the population, which was consistent with our working hypothesis and earlier studies (Antanaviciute et al., 2015; Bringhurst, Wilhelm, & Voth, 1966, 1968; Cockerton et al., 2019; Maas et al., 1989; Shaw et al., 1996; Vining et al., 2015). The absence of significant marker‐trait associations could mean that large‐effect QTL were not segregating in the training population, that sample size and statistical power were insufficient to identify genotype‐to‐phenotype associations, or that the frequencies of minor alleles in linkage disequilibrium with favorable QTL alleles were too low to identify statistically significant associations (Gibson, 2012, 2018; Simons, Bullaughey, Hudson, & Sella, 2018; Young, 2019; Zaitlen et al., 2013).

### 3.7 Genomic selection for resistance to Verticillium wilt

**FIGURE 6:**
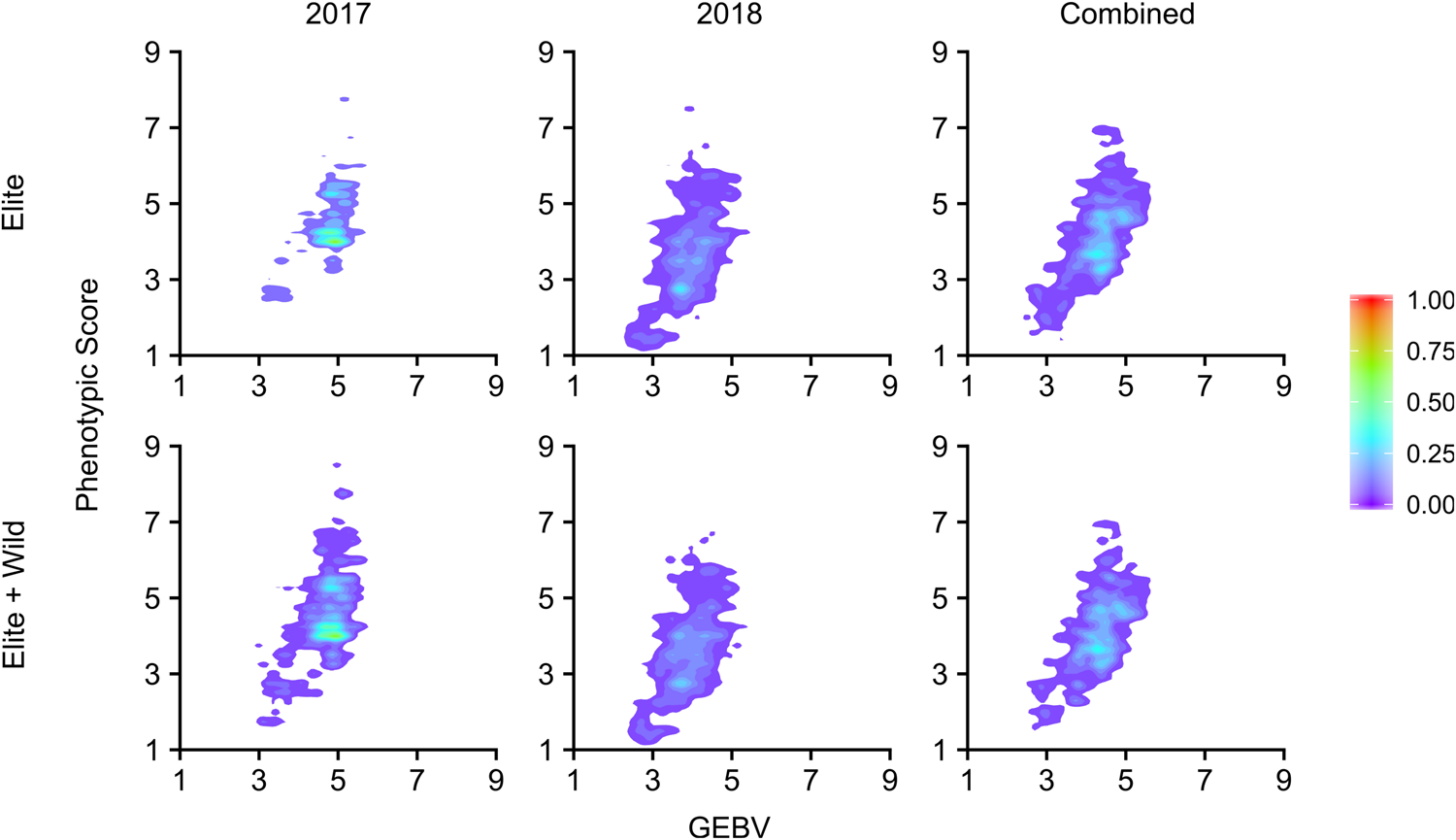
Kernel density distributions for genomic‐BLUP estimated GEBVs plotted against observed phenotypic scores for Verticillium wilt resistance in a strawberry population comprised of 347 *F. × ananassa*, 20 *F. chiloensis*, and 21 *F. virginiana* accessions. GEBV estimates for each distribution were produced by cross‐validation with *k* = 10,000 replicates, where GEBVs for 20% of the accessions were estimated from GEBVs for 80% of the accessions randomly sampled from the population. The training population was phenotyped in 2017 and 2018 field experiments in Davis, California and genotyped with a 50K SNP array (Hardigan et al., 2020). GEBVs were estimated using genomic‐BLUP for the elite + wild training population (*n* = 388) and elite (*F. × ananassa*) training population (*n* = 347) within and between years

Genomic prediction accuracies were virtually identical for whole‐genome regression analyses performed by modeling the response variable as continuous or ordinal (Table 1; Figure 6). The mean accuracies for continuous and ordinal analyses (108 combinations) were identical (0.45). Similarly, the accuracy ranges were virtually identical for continuous (0.38–0.52) and ordinal (0.31–0.53) analyses (Table 1). The correlation between accuracies estimated from continuous and ordinal response variable analyses was 0.84 (*p* < .0001; Table 1). Henceforth, results for the continuous response variable are discussed unless noted otherwise. Our conclusions apply equally well to the ordinal response variable analyses (Table 1; Figure 6; Supplemental Figure S5).

**TABLE 1:** Accuracy of genomic predictions for resistance to Verticillium wilt in a training population comprised of 347 *F. × ananassa*, 20 *F. chiloensis*, and 21 *F. virginiana* accessions. These accessions were phenotyped in 2017 and 2018 field experiments in Davis, California and genotyped with a 50K SNP array (Hardigan et al., 2020). GEBVs were estimated for the complete (elite + wild) training population (*n* = 388) and *F. × ananassa* (elite) training population (*n* = 347) using genomic‐BLUP (G‐BLUP), the Bayesian Lasso (BL), and reproducing kernel Hilbert spaces (RKHS) whole‐genome regression methods implemented in *rrBLUP* and *BGLR* (Endelman, 2011; Pérez & de los Campos, 2014). GEBVs were estimated within and between years by modeling the response variables as continuous (normal) or categorical (ordinal)

	G‐BLUP			RKHS			BL		
Source	2017	2018	Combined	2017	2018	Combined	2017	2018	Combined
Normal	r(ȳ,GEBV)								
Elite + Wild									
2017	0.41	0.38	0.45	0.42	0.38	0.46	0.42	0.38	0.46
2018	0.38	0.46	0.48	0.39	0.45	0.49	0.39	0.45	0.49
Combined	0.41	0.45	0.49	0.42	0.44	0.50	0.42	0.44	0.50
Elite									
2017	0.49	0.40	0.50	0.49	0.39	0.50	0.49	0.39	0.50
2018	0.43	0.47	0.51	0.44	0.45	0.50	0.44	0.45	0.50
Combined	0.48	0.45	0.53	0.48	0.44	0.52	0.48	0.44	0.52
Ordinal	r(ȳ,GEBV)								
Elite + Wild									
2017	0.42	0.36	0.44	0.42	0.36	0.44	0.42	0.38	0.46
2018	0.42	0.44	0.50	0.43	0.44	0.50	0.31	0.47	0.50
Combined	0.43	0.41	0.48	0.44	0.41	0.48	0.43	0.44	0.50
Elite									
2017	0.45	0.37	0.47	0.49	0.37	0.48	0.50	0.39	0.50
2018	0.41	0.44	0.48	0.49	0.44	0.52	0.46	0.47	0.53
Combined	0.45	0.42	0.49	0.49	0.41	0.51	0.50	0.44	0.53

Genomic prediction accuracies were virtually identical for the three whole‐genome regression methods (G‐BLUP, RKHS, and BL), which were applied to elite and elite + wild training populations within and between years, e.g., accuracies ranged from 0.52–0.53 for the three whole‐genome regression (WGR) methods when both years of data for the elite training population were analyzed (Table 1; Figure 6; Supplemental Figure S5). These were the highest genomic prediction accuracies achieved in our study. RKHS, BL, and related WGR methods are predicted to produce superior results relative to G‐BLUP when the effects of the underlying causative loci are non‐uniform and non‐additive (Gianola, 2013; Gianola, Fernando, & Stella, 2006, 2009). The superiority of WGR methods for GEBV estimation, however, has been difficult to predict *a priori* because the underlying genetic architectures of quantitative traits are typically unknown, a problem that has commonly been approached by empirical head‐to‐head comparisons of prediction accuracies for different WGR methods (Crossa et al., 2013; de los Campos et al., 2010; Gianola et al., 2006; González‐Camacho et al., 2012; Heslot, Yang, Sorrells, & Jannink, 2012; Pérez‐Rodríguez et al., 2012). The WGR methods we tested yielded virtually identical results, with G‐BLUP yielding the most accurate predictions (Gianola, de los Campos, Hill, Manfredi, & Fernando, 2009; Table 1; Figure 6).

**FIGURE 7:**
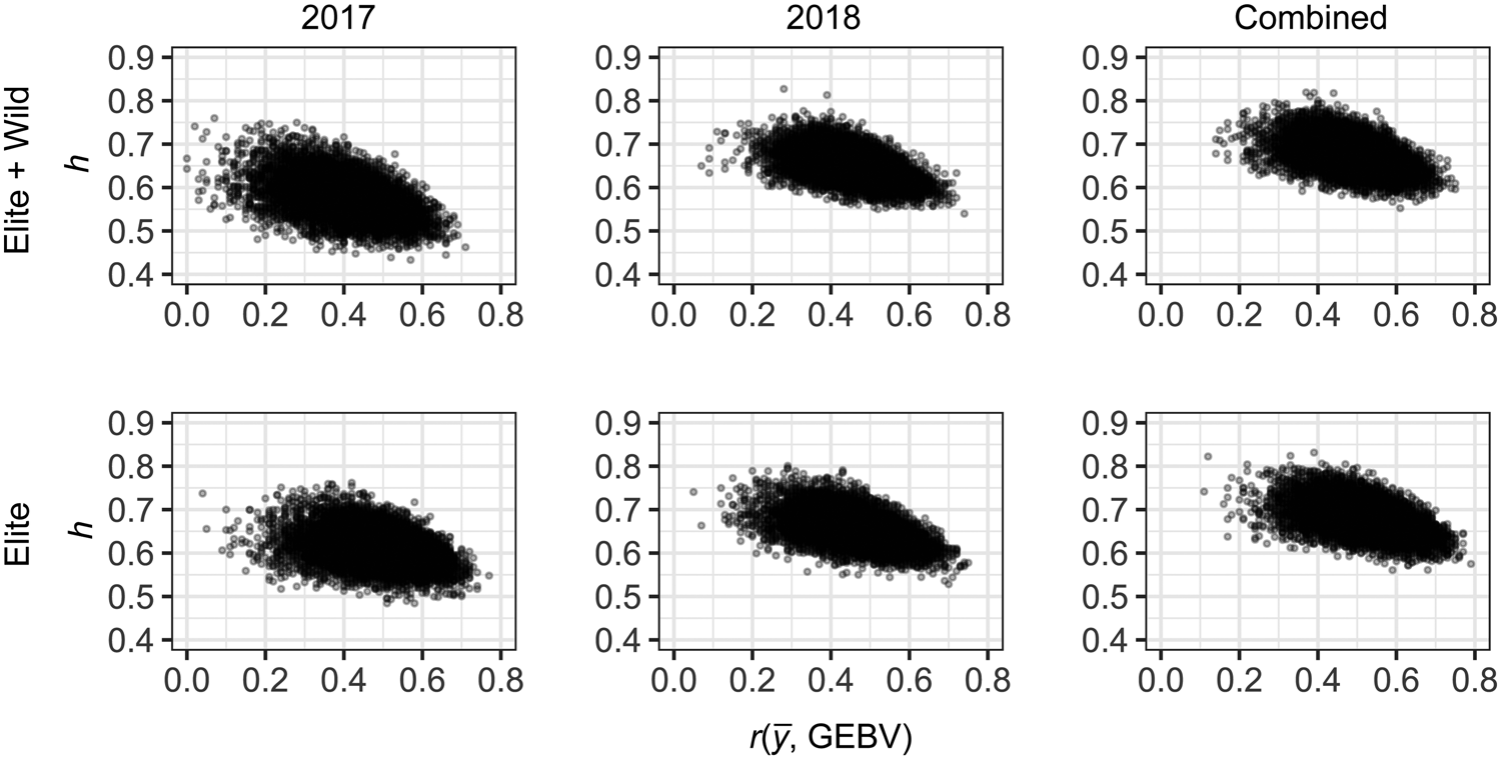
G‐BLUP genomic prediction accuracy and narrow‐sense heritability (*h* ^2^) estimates obtained by cross‐validation with *k* = 10,000 replicates, where *r*(*ȳ*, GEBV) is the genomic prediction accuracy, *h* is the square‐root of *h* ^2^, accessions were randomly sampled from the training population, and GEBVs for 20% of the accessions were estimated from GEBVs for 80% of the accessions. G‐BLUP GEBVs were estimated for elite + wild (*n* = 388) and elite (*n* = 347) training populations within and between years

To estimate the accuracy of genomic selection relative to phenotypic selection (*r_E_*), estimates of the correlation between *ȳ* and GEBV estimates (*r_GS_*) were divided by estimates of the square root of the narrow‐sense genetic heritability (Crossa et al., 2014). For the elite + wild training population, the *r_E_* estimates from G‐BLUP were 0.71 for 2017, 0.71 for 2018, and 0.74 between years (Figure 7). For the elite training population, the *r_E_* estimates from G‐BLUP were 0.80 for 2017, 0.72 for 2018, and 0.78 between years (Figure 7). These were the highest relative accuracies achieved in our study and suggest that genomic selection, with adequate training (phenotyping and genotyping), has significant potential for identifying superior genotypes without phenotyping, albeit with periodic retraining (Crossa et al., 2013, 2014; Goddard & Hayes, 2007; Hayes et al., 2009; Heffner, Sorrells, & Jannink, 2009, 2010; Heslot et al., 2012, 2015; Hickey et al., 2014; Jannink, Lorenz, & Iwata, 2010; Poland & Rutkoski, 2016).

The addition of the 41 wild ecotypes to the training population only slightly decreased genomic prediction accuracies (Table 1). For the between‐year analyses, accuracies ranged from 0.49–0.50 for the elite + wild training population versus 0.52–0.53 for the elite training population. We anticipated some loss in accuracy because of increased genetic divergence between elite and wild accessions (Figure 4), analogous to the challenges encountered in predicting breeding values across breeds or heterotic groups (Dekkers & Van der Werf, 2007; Hidalgo et al., 2015; Ibánẽz‐Escriche, Fernando, Toosi, & Dekkers, 2009; Massman, Jung, & Bernardo, 2013; Technow et al., 2014; Veroneze et al., 2015; Windhausen et al., 2012). Nevertheless, our results suggest that breeding values can be accurately predicted in genetically diverse strawberry populations, perhaps because the genomes of *F. × ananassa* lineages are admixtures of the genomes of the wild octoploid progenitors (Hardigan et al., 2018, 2020). Although breeding has significantly restructured genetic diversity, cultivated strawberry has retained much of the allelic diversity found in the wild octoploid progenitors (Hardigan et al., 2018, 2020).

Genomic prediction accuracies were slightly greater when GEBVs were estimated from both years as opposed to one year of data, as predicted from differences in narrow‐sense heritability estimates (Table 1; Figure 6; Supplemental Figure S5). The decreases in accuracy for within and between year analyses, however, were modest, e.g., for G‐BLUP analyses of the elite training population, accuracies were 0.49 in 2017, 0.47 in 2018, and 0.53 for between years. Amongst the combinations of WGR methods and response variable distributions (continuous or ordinal), we found that accuracies were lower when one year was used for predicting the other year, e.g., for G‐BLUP analyses of the elite training population, predicting 2018 from 2017 and vice versa yielded accuracies of 0.40 and 0.43 as opposed to within‐year accuracies of 0.47 and 0.49 (Table 1; Supplemental Figure S6). We did not observe a significant decrease in accuracy in the elite training population with any of the WGR methods when the data from a single year was used to predict GEBVs between years, e.g., for G‐BLUP, the accuracies for 2017 or 2018 data predicting between years were 0.50 and 0.51, whereas the accuracy for the between year data predicting between years was 0.53 (Table 1).

One of the goals of our study was to assess the feasibility of increasing genetic gains in breeding for resistance to Verticillium wilt by applying genomic selection (Crossa et al., 2013; Heslot et al., 2012; Poland & Rutkoski, 2016). Shortening selection cycles is a common approach for increasing the genetic gain of genomic selection relative to phenotypic selection. With no historic phenotypic data on which to base predictions or assess year and genotype‐by‐year effects, we tested clonally replicated accessions over two years, which necessitated the clonal propagation of accessions in high‐elevation nurseries, in addition to replicated testing of artificially inoculated bare‐root plants in field experiments. We observed a negligible increase in broad‐sense heritability with the addition of a second year of phenotyping (0.73 in 2017, 0.71 in 2018, and 0.76 between years); however, the differences in narrow‐sense genomic heritability were more substantial (0.33 in 2017, 0.42 in 2018, and 0.45 between years). Genomic prediction accuracies were only slightly greater when both years versus one year of data were used for estimating GEBVs (Table 1; Supplemental Figure S5). Our results suggest that genetic gains can be increased by shortening the selection cycle in strawberry (using one year of phenotyping for training genomic prediction models). The minimal gain in accuracy achieved with multiple years of testing appears to be outweighed by the increase in genetic gain that could be achieved by shortening the length of the selection cycle (Table 1; Supplemental Figure S5). The first cycle of selection would include phenotyping and genotyping (training), which could be combined with a second cycle of selection without phenotyping (genotyping only). The downside risk associated with a single year of phenotyping could be mitigated by increasing the scale of testing and maintaining a high selection intensity (selecting and advancing a larger number of accessions), another approach enabled by genomic selection (Heslot, Jannink, & Sorrells, 2015; Jannink et al., 2010). Our results suggest that genetic gains can be further increased by increasing throughput (increasing the number of genotyped accessions that are not phenotyped). Genomic selection in the seedling (juvenile) stage without phenotyping adult plants could further shorten the length of a cycle of selection and reduce clonal propagation costs, thereby significantly reducing the cost per selection cycle (Poland & Rutkoski, 2016). Our study, however, does not shed light on the effectiveness of the seedling approach, a problem that warrants further study.

GEBVs for accessions in the susceptible range (4 to 9) were slightly more shrunk than those in the resistant range (1 to 3), particularly in the elite training population. The *ȳ* × GEBV density distributions were crescent‐shaped with accessions in the lower third of the distribution being shrunk slightly less than accessions in the upper two‐thirds of the distribution (Figure 6; Supplemental Figure S6). We concluded that breeding values of resistant to highly resistant accessions (the targets of selection) were more accurately predicted than breeding values of moderately to highly susceptible accessions. The visual ratings may not have differentiated the severity of disease symptoms among moderately to highly susceptible accessions as well as among resistant to highly resistant accessions. Our results suggest that the visual ratings effectively identified the most resistant accessions, which are of primary importance in breeding for resistance to this pathogen.

## 4 CONCLUSIONS

The development of Verticillium wilt resistant cultivars has been a persistent challenge in strawberry, as evidenced by the high frequency of moderately to highly susceptible cultivars across the globe. There were time periods over the last 165 years, particularly following the introduction and subsequent widespread adoption of soil fumigation in the late 1950s, when selection for resistance to Verticillium wilt was relaxed and deprioritized. The resultant decrease in selection pressure and negative genetic gains over that period highlight the urgency of intensifying selection for resistance to this pathogen. With the reliance on soil fumigation decreasing and the importance of organic production increasing worldwide, the need for Verticillium wilt resistant cultivars has never been greater. Our study shows that heirloom cultivars and wild ecotypes may be the strongest novel sources of resistance to this pathogen. Although these exotic germplasm sources undoubtedly carry novel favorable alleles for resistance to Verticillium wilt, they are several decades of breeding and hundreds of thousands of recombination events removed from the horticulturally superior cultivars that dominate the production landscape today. Hence, the most promising exotic donors of favorable alleles for resistance to Verticillium wilt undoubtedly harbor a high frequency of unfavorable alleles for other horticulturally important traits. Consequently, the introduction of novel favorable alleles for Verticillium wilt resistance into modern cultivars poses a significant technical challenge. Genomic selection has the potential to accelerate that process and outperform phenotypic selection.

## AUTHOR CONTRIBUTIONS

DDP planned, designed, and organized the field experiments, phenotyped the population, performed statistical analyses, analyzed data, and developed the supporting materials. SJK conceived and designed the study and analyzed data. SJK, GSC, and DDP identified and selected cultivars, ecotypes, and other accessions for the study. MAH developed the 50K SNP array. MAH, DDP, and RAF produced, analyzed, and curated the genotypic data. GSC organized and managed the populations, field experiments, and propagation of clonal plant materials. TRG provided guidance on artificial inoculation protocols and pathogen inoculum. PMH identified the vegetative compatibility group of the pathogen isolate. All authors read and approved the final manuscript.

## CONFLICT OF INTEREST DISCLOSURE

The authors declare that there were no conflicts of interest.

## Supporting information

Supporting Material

Supporting Material

Supporting Material

Supporting Material

**Supplemental Fig. S1**. The physical locations of 981 out of 984 strawberry accessions mapped using latitude and longitude coordinates (the latter were unavailable for three accessions). Coordinates identify the breeding stations where *F. × ananassa* cultivars and accessions were developed or habitats where wild *F. chiloensis* and *F. virginiana* ecotypes were originally collected.

**Supplemental Fig. S2**. Least‐square means (LSMs) for Verticillium wilt resistance are plotted for 890 strawberry accessions by latitude (upper panel) and longitude (lower panel). Geographic coordinates identify the physical locations of breeding stations where *F. × ananassa* cultivars and accessions were developed or habitats wild *F. chiloensis* and *F. virginiana* ecotypes were originally collected. LSMs were estimated from four replications/accession in the 2017 field study in Davis, CA.

**Supplemental Fig. S3**. The distribution of 35,144 SNP markers in 0.25 Mb windows across the octoploid reference genome (Edger et al., 2019). SNP markers were genotyped with a 50K SNP array (Hardigan et al., 2020).

**Supplemental Fig. S4**. Manhattan plots displaying GWAS‐estimated ‐log_10_ *p*‐values for Verticillium wilt resistance in 2017 and 2018 in a training population comprised of 347 *F. × ananassa*, 20 *F. chiloensis*, and 21 *F. virginiana* accessions. The training population was phenotyped in 2017 and 2018 field experiments in Davis, California and genotyped with a 50K SNP array (Hardigan et al., 2020). GWAS statistics were estimated for resistance phenotypes for accession years using physical positions in an octoploid reference genome and genotypes for 35,144 SNPs. Significance thresholds (dashed horizontal lines) were calculated by permutation (‐log_10_ *p*‐values were 5.48 for 2017 and 5.02 for 2018).

**Supplemental Fig. S5**. Kernel density distributions for genomic‐BLUP estimated GEBVs for Verticillium wilt resistance in a strawberry training population estimated by cross‐validation with 10,000 replicates. The training population was phenotyped in 2017 and 2018 field experiments in Davis, California and genotyped with a 50K SNP array. GEBVs were estimated by genomic‐BLUP for the complete training population (n = 388) using a single year (2017 or 2018) or both years of phenotypic data in all possible combinations.

## Data Availability

The raw phenotypic and genotypic data and other supporting data for this study were made available as supplemental materials.
