# Supplementary material for: Accuracy of genomic selection and long‐term genetic gain for resistance to Verticillium wilt in strawberry
Source: Plant Genome. 2020 Sep 24;13(3):e20054. doi: 10.1002/tpg2.20054 (PMC12807005; doi:10.1002/tpg2.20054)
Supplement: Supplementary file 2 [file TPG2-13-e20054-s003.pdf]

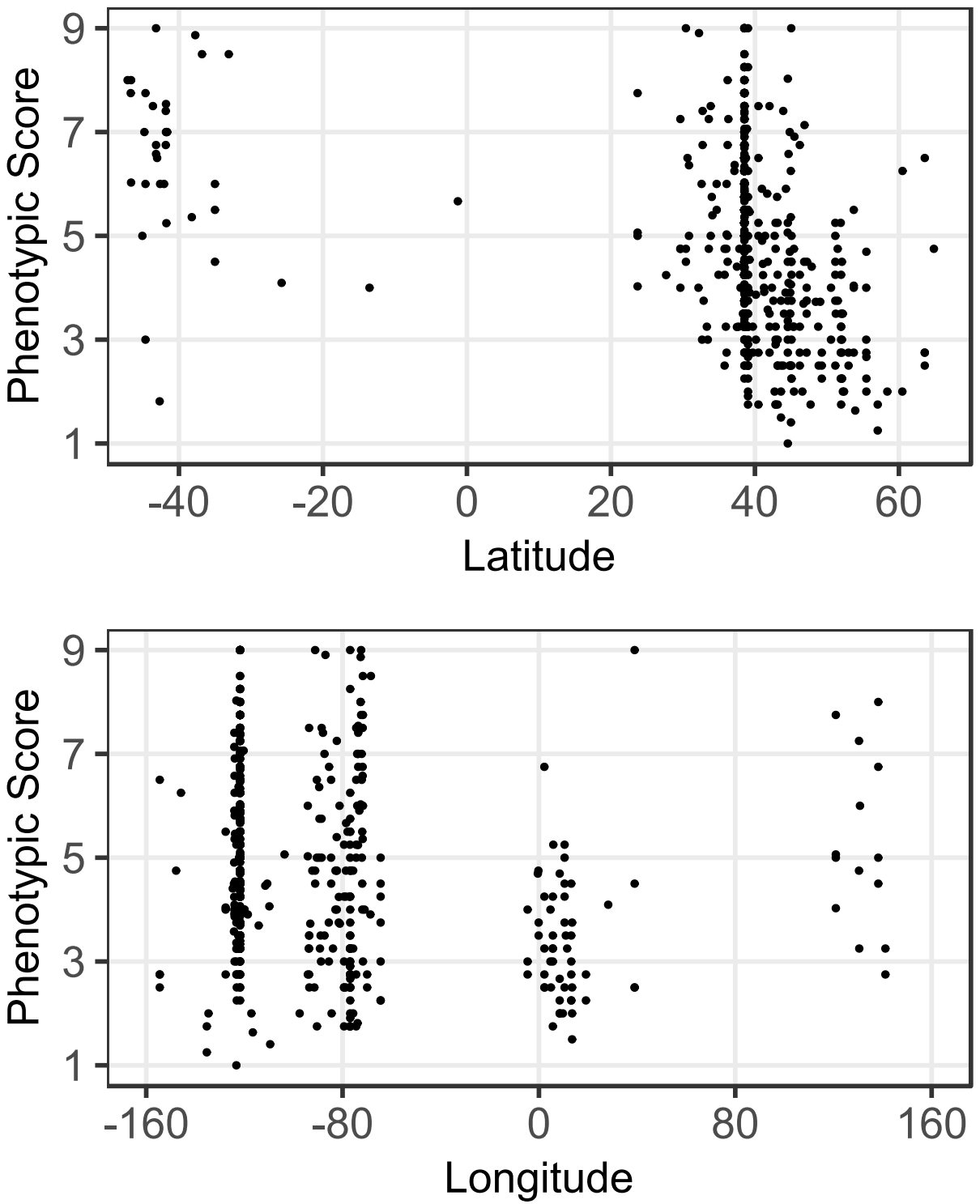

Phenotypic Score
9
7
5
3
1
-40
-20
0
20
40
60
Latitude
Phenotypic Score
9
7
5
3
1
-160
-80
0
80
160
Longitude
