# Supplementary material for: Accuracy of genomic selection and long‐term genetic gain for resistance to Verticillium wilt in strawberry
Source: Plant Genome. 2020 Sep 24;13(3):e20054. doi: 10.1002/tpg2.20054 (PMC12807005; doi:10.1002/tpg2.20054)
Supplement: Supplementary file 4 [file TPG2-13-e20054-s006.pdf]

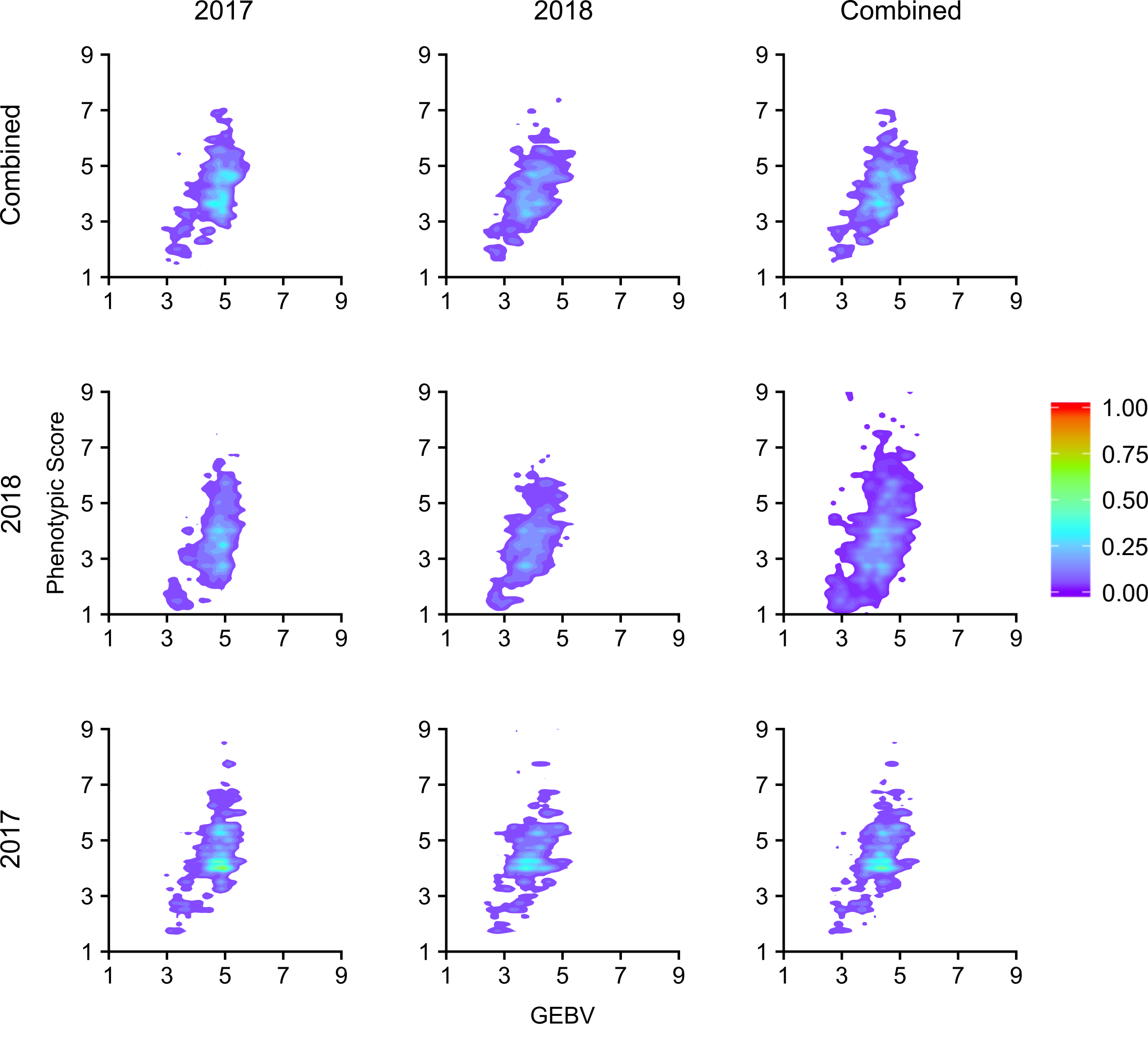

2017
2018
Combined
Combined
2018
2017
Phenotypic Score
GEBV
1.00
0.75
0.50
0.25
0.00
1
3
5
7
9
