# Supplementary material for: Accuracy of genomic selection and long‐term genetic gain for resistance to Verticillium wilt in strawberry
Source: Plant Genome. 2020 Sep 24;13(3):e20054. doi: 10.1002/tpg2.20054 (PMC12807005; doi:10.1002/tpg2.20054)
Supplement: Supplementary file 5 [file TPG2-13-e20054-s008.pdf]

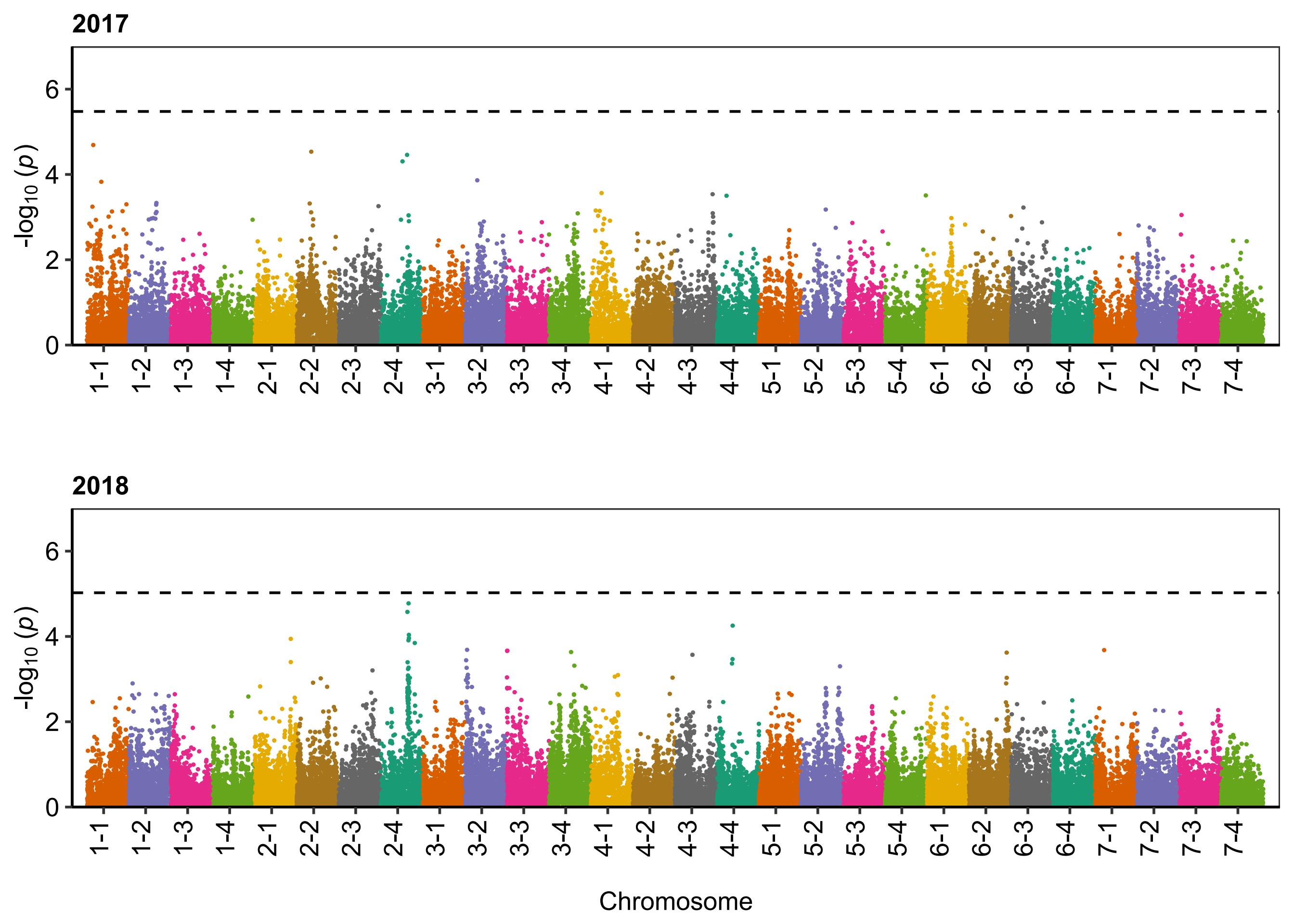

2017
2018
-log10 (p)
0
2
4
6
1-1
1-2
1-3
1-4
2-1
2-2
2-3
2-4
3-1
3-2
3-3
3-4
4-1
4-2
4-3
4-4
5-1
5-2
5-3
5-4
6-1
6-2
6-3
6-4
7-1
7-2
7-3
7-4
Chromosome
