# Supplementary material for: Accuracy of genomic selection and long‐term genetic gain for resistance to Verticillium wilt in strawberry
Source: Plant Genome. 2020 Sep 24;13(3):e20054. doi: 10.1002/tpg2.20054 (PMC12807005; doi:10.1002/tpg2.20054)
Supplement: Supplementary file 6 [file TPG2-13-e20054-s009.docx]

**SUPPLEMENTAL MATERIALS FOR: “Accuracy of Genomic Selection and Long-Term Genetic Gain for Resistance to Verticillium Wilt in Strawberry”**

Dominique D.A. Pincot^1^, Michael A. Hardigan^1^, Glenn S. Cole^1^, Randi A. Famula^1^, Peter M. Henry^2^, Thomas R. Gordon^3^, and Steven J. Knapp^1^*

^1^Department of Plant Sciences, University of California, One Shields Avenue, Davis, California 95616; ^2^United States Department of Agriculture, 1636 E. Alisal Street, Salinas, California 93905; ^3^Department of Plant Pathology, University of California, One Shields Avenue, Davis, California 95616

**Supplemental File S1**. Verticillium wilt resistance phenotypes (least-square means estimated from four replications/accession) and passport information for 984 strawberry accessions screened in 2017 and 2018 field experiments in Davis, California. The passport information includes geographic origin, year of origin, country of origin, taxonomic classification, UCD Strawberry Germplasm Collection identification numbers for UCD accessions (*n* = 673), plant introduction (PI) numbers for USDA accessions (*n* = 311), and aliases, e.g., cultivar names. The geographic coordinates (latitudes and longitudes) recorded in the database identify the physical locations of breeding stations where *F. × ananassa* cultivars and accessions were developed or habitats where wild *F. chiloensis* and *F. virginiana* ecotypes were originally collected.

**Supplemental File S2.** Verticillium wilt resistance phenotypes collected from four replications/accession/year among 984 strawberry accessions screened in 2017 and 2018 field experiments in Davis, California. The accessions in the study population are identified by UCD identification (UCD ID) or USDA Plant Introduction (PI) numbers.

**Supplemental File S3.** Coded genotypes for 35,144 SNP markers among 388 strawberry accessions in the training population. Accessions are identified by UCD identification (UCD ID) or USDA Plant Introduction (PI) numbers. SNP markers were genotyped with a 50K SNP array. SNP genotypes are coded in a -1, 0, 1 format, which corresponds to homozygote, heterozygote, and opposite homozygote, respectively, with missing data coded NA. Chromosome numbers and physical positions (Mb) of SNP markers follow the nomenclature of Edger et al. (2019) and Hardigan et al. (2020).

**Supplemental Fig. S1.** The physical locations of 981 out of 984 strawberry accessions mapped using latitude and longitude coordinates (the latter were unavailable for three accessions). Coordinates identify the breeding stations where *F. × ananassa* cultivars and accessions were developed or habitats where wild *F. chiloensis* and *F. virginiana* ecotypes were originally collected.

**Supplemental Fig. S2.** Least-square means (LSMs) for Verticillium wilt resistance are plotted for 890 strawberry accessions by latitude (upper panel) and longitude (lower panel). Geographic coordinates identify the physical locations of breeding stations where *F. × ananassa* cultivars and accessions were developed or habitats wild *F. chiloensis* and *F. virginiana* ecotypes were originally collected. LSMs were estimated from four replications/accession in the 2017 field study in Davis, CA.

**Supplemental Fig. S3.** The distribution of 35,144 SNP markers in 0.25 Mb windows across the octoploid reference genome (Edger et al., 2019). SNP markers were genotyped with a 50K SNP array (Hardigan et al., 2020).

**Supplemental Fig. S4.** Manhattan plots displaying GWAS-estimated -log_10_ *p*-values for Verticillium wilt resistance in 2017 and 2018 in a training population comprised of 347 *F. × ananassa*, 20 *F. chiloensis*, and 21 *F. virginiana* accessions. The training population was phenotyped in 2017 and 2018 field experiments in Davis, California and genotyped with a 50K SNP array (Hardigan et al., 2020). GWAS statistics were estimated for resistance phenotypes for accession years using physical positions in an octoploid reference genome and genotypes for 35,144 SNPs. Significance thresholds (dashed horizontal lines) were calculated by permutation (-log_10_ *p*-values were 5.48 for 2017 and 5.02 for 2018).

**Supplemental Fig. S5.** Kernel density distributions for genomic-BLUP estimated GEBVs for Verticillium wilt resistance in a strawberry training population estimated by cross-validation with 10,000 replicates. The training population was phenotyped in 2017 and 2018 field experiments in Davis, California and genotyped with a 50K SNP array. GEBVs were estimated by genomic-BLUP for the complete training population (*n* = 388) using a single year (2017 or 2018) or both years of phenotypic data in all possible combinations.
